# Telemedicine in an Outpatient Arthroplasty Setting During the COVID-19 Pandemic: Early Lessons from New York City

**DOI:** 10.1177/1556331620972659

**Published:** 2021-02-21

**Authors:** Drake G. LeBrun, Christina Malfer, Mallory Wilson, Kaitlin M. Carroll, Victoria Wang, MS, David J. Mayman, Michael B. Cross, Michael M. Alexiades, Seth A. Jerabek, Fred D. Cushner, Jonathan M. Vigdorchik, Steven B. Haas, Michael P. Ast

**Affiliations:** 1 Hospital for Special Surgery, New York, NY, USA

**Keywords:** telemedicine, total knee arthroplasty, total hip arthroplasty, COVID-19, outpatient arthroplasty

## Abstract

*Background:* The early months of the coronavirus disease 19 (COVID-19) pandemic in New York City led to a rapid transition of non-essential in-person health care, including outpatient arthroplasty visits, to a telemedicine context. *Questions/Purposes:* Based on our initial experiences with telemedicine in an outpatient arthroplasty setting, we sought to determine early lessons learned that may be applicable to other providers adopting or expanding telemedicine services. *Methods:* A cross-sectional study was performed by surveying all patients undergoing telemedicine visits with 8 arthroplasty surgeons at 1 orthopedic specialty hospital in New York City from April 8 to May 19, 2020. Descriptive statistics were used to analyze demographic data, satisfaction with the telemedicine visit, and positive and negative takeaways. *Results:* In all, 164 patients completed the survey. The most common reasons for the telemedicine visit were short-term (less than 6 months), postoperative appointment (*n* = 88; 54%), and new patient consultation (*n* = 32; 20%). A total of 84 patients (51%) noted a reduction in expenses versus standard outpatient care. Several positive themes emerged from patient feedback, including less anxiety and stress related to traveling (*n =* 82; 50%), feeling more at ease in a familiar environment (*n =* 54; 33%), and the ability to assess postoperative home environment (*n =* 13; 8%). However, patients also expressed concerns about the difficulty addressing symptoms in the absence of an in-person examination (*n =* 28; 17%), a decreased sense of interpersonal connection with the physician (*n =* 20; 12%), and technical difficulties (*n =* 14; 9%). *Conclusions:* Patients were satisfied with their telemedicine experience during the COVID-19 pandemic; however, we identified several areas amenable to improvement. Further study is warranted.

## Introduction

The coronavirus disease 19 (COVID-19) pandemic led to an unprecedented transformation in the delivery of health care services worldwide. As a result of the unique infection control challenges posed by COVID-19, the US Centers of Disease Control and Prevention (CDC) recommended that health care systems “adjust their standard approaches . . . to reduce the need to provide in-person care to minimize risk to patients and [health care providers]” [4]. In response to these recommendations and other social distancing guidelines from state and local governments, many providers have had to rapidly adopt or expand telemedicine practices [2,11]. With forecasts suggesting that social distancing practices will continue in 2021 [20], physicians should be prepared to continue using telemedicine platforms for the foreseeable future.

Telemedicine platforms—previously underused in the orthopedic community [16]—have allowed orthopedic providers to continue seeing patients while adhering to social distancing guidelines. Preliminary reports from new or expanded telemedicine practices during the pandemic in fracture clinics [21], outpatient rehabilitation centers [17], upper extremity postoperative clinics [7], and general orthopedic clinics [13,19] have highlighted several advantages of telemedicine, as well as challenges associated with the accelerated adoption of telemedicine platforms. Total joint arthroplasty represents a unique challenge in the context of telemedicine, as patients undergoing total joint arthroplasty are often older, have comorbidities that put them at higher risk of complications related to COVID-19, and may be more reluctant to try telehealth given concerns that it may be too technical and confusing [1,14,23]. Nonetheless, with appropriate coordination, telemedicine can allow orthopedic providers to continue seeing patients in a total joint arthroplasty context while minimizing in-person exposure [18].

During the early phases of the COVID-19 pandemic (March and April 2020) at our orthopedic specialty hospital in New York City, telemedicine was rapidly expanded to continue providing care to new and follow-up patients [8]. To evaluate our initial experiences with telemedicine in total joint arthroplasty patients, we developed a survey for patients to better understand their early experiences and how our telemedicine practices can be improved.

The purposes of this study were to (1) describe initial patient experiences with our telemedicine platform in an outpatient arthroplasty setting and (2) explore early lessons learned that may be applicable to other providers adopting or expanding their telemedicine practices.

## Methods

A cross-sectional study was performed by surveying all patients undergoing telemedicine visits with 8 arthroplasty surgeons at 1 orthopedic specialty hospital in New York City from April 8 to May 19, 2020. The survey included questions regarding baseline demographics, prior experience with telemedicine, satisfaction with the telemedicine visit, difficulties with the visit, logistics of the visit (e.g., how the patient accessed the visit), positive and negative aspects of the visit (including both prompts and free-text options), the financial effects of telemedicine, and how the patient would compare the telemedicine experience with the standard outpatient visit. The full survey is included in Supplemental Figure 1.

To develop the survey, a literature review on telemedicine surveys was first performed. A set of questions was derived from prior surveys in the literature [3,5] and questions were modified as needed to be relevant to an arthroplasty context. We designed the survey with 4 foci: (1) demographic/clinical, (2) logistics of accessing the telemedicine visit, (3) patient satisfaction and engagement, and (4) positive and negative experiences. Of note, the survey was not validated. A description of the survey and instructions for how to perform the survey were e-mailed to all patients following their telemedicine visit. If radiographs were deemed necessary by the surgeon, they were obtained at satellite radiography facilities and reviewed during the visit by the surgeon with the patient.

Descriptive statistics were used to analyze patient responses. Free-text and categorical responses for positive and negative takeaways were consolidated and reported together. All calculations were performed using STATA 16.0 (StataCorp, College Station, TX, USA). The study was approved by the Institutional Review Board.

## Results

In all, 445 patient visits were initially included. Of these, 122 were excluded because they were either repeat visits (*n =* 93), did not have contact information in the electronic medical record (*n =* 16), or needed an interpreter (*n =* 13). Therefore, out of 323 patients, 164 (51%) completed the survey (Table 1). The most common reasons for the telemedicine visit were short-term (<6 months) postoperative appointment (*n =* 88; 54%) and new patient consultation (*n =* 32; 20%). In all, 122 (74%) had never used telemedicine before and 46 (28%) needed assistance from someone else with accessing the telemedicine visit (Table 2). Overall, 155 patients (96%) were slightly to very satisfied with the transition process to telemedicine, while 7 patients (4%) were slightly to very dissatisfied (Table 3). A total of 84 patients (51%) noticed a reduction in expenses using telemedicine versus normal outpatient care. Of these, 83 (99%) noted a decrease in travel costs, 14 (17%) noted a decrease in work costs, and 5 (6%) noted a decrease in medical costs. Compared with standard outpatient treatment, 128 patients (78%) had an experience that was good, excellent, or the best imaginable, while 31 patients (19%) noted that their experience was OK or poor. In all, 111 patients (68%) would consider continuing telemedicine in addition to outpatient treatment.

**Table 1.** Demographic and clinical characteristics of surveyed patients.

	*n*	%
Age (y)		
26–40	2	1.2
41–65	88	53.7
66–80	69	42.1
>80	5	3.0
Surgery^a^		
Follow-up (no surgery)	9	5.5
New patient (no surgery)	24	14.6
Unicompartmental Knee Arthroplasty (UKA)	5	3.0
Total Knee Arthroplasty (TKA)	60	36.6
Total Hip Arthroplasty (THA)	47	28.7
Revision TKA	9	5.5
Revision THA	6	3.7
Employment^a^		
Retired	69	42.1
Full-time	66	40.2
Part-time	14	8.5
Unemployed	8	4.9
Medical leave	6	3.7
Prior telemedicine experience		
Yes	42	25.6
No	122	74.4
Reason for visit^a^		
Short-term follow-up (<6 mo)	88	53.7
Long-term follow-up (6+ mo)	5	3.0
Follow-up (unknown duration)	8	4.9
New patient	32	19.5
New issue unrelated to prior surgery	10	6.1
Physical therapy	8	4.9
Preoperative planning	3	1.8

a Counts for some variables may not add to 164 due to missing responses (Surgery, *n* = 4; Employment, *n* = 1; Reason for visit, *n* = 10).

**Table 2.** Logistics of telemedicine visit among surveyed patients.

	*n*	%
Device used to access telemedicine visit		
Computer	79	48.2%
Phone	47	28.7%
Tablet	24	14.6%
More than 1 device	14	8.5%
Needed assistance with accessing the telemedicine visit		
Yes	46	28.0%
No	114	69.5%
No response	4	2.4%
Difficulties during the telemedicine visit		
Yes	20	12.2%
No	139	84.8%
No response	5	3.0%

**Table 3.** Responses to patient experience questions.

	*n*	%
How satisfied are you with the support you received during the transition process to telemedicine at HSS?		
Very satisfied	117	71.3
Satisfied	34	20.7
Slightly satisfied	4	2.4
Slightly dissatisfied	3	1.8
Dissatisfied	1	0.6
Very dissatisfied	3	1.8
No response	2	1.2
Did you notice a reduction in expenses using telemedicine vs. outpatient care at HSS?		
Yes	84	51.2
No	67	40.9
No response	13	7.9
Compared with in-person visits, how would you rate your personal engagement and attentiveness to your own health and recovery?		
Better or much better	33	20.1
Same	107	65.2
Worse or much worse	17	10.4
No response	7	4.3
Did you find an increase in convenience and flexibility with virtual follow-up care compared with outpatient treatment?		
Yes	85	51.8
No	19	11.6
Neutral	53	32.3
No response	7	4.3
Did you experience the same degree of attention and interaction with your physician as you would expect in the examination room?		
Yes	108	65.9
No	21	12.8
Neutral	31	18.9
No response	4	2.4
Do you feel that you were able to discuss all or most of your concerns during the consultation?		
Yes	142	86.6
No	9	5.5
Neutral	10	6.1
No response	3	1.8
Overall, compared with standard outpatient treatment, how would you describe your telemedicine experience?		
Best imaginable	10	6.1
Excellent	78	47.6
Good	40	24.4
Ok	29	17.7
Poor	2	1.2
No response	5	3.0
Would you consider continuing telemedicine care in addition to outpatient treatment at HSS?		
Yes	111	67.7
No	11	6.7
Maybe	40	24.4
No response	2	1.2

Several positive themes emerged in our initial experience (Table 4), including less anxiety and stress related to traveling (*n =* 82; 50%), feeling more at ease in a familiar environment (*n =* 54; 33%), a longer appointment time (*n =* 16; 10%), and the ability to assess postoperative home environment (*n =* 13; 8%). However, patients also cited concerns about the difficulty addressing symptoms in the absence of an in-personal examination (*n =* 28; 17%), a decreased sense of interpersonal connection with the physician (*n =* 20; 12%), technical difficulties (*n =* 14; 9%), a shorter appointment time (*n =* 6; 4%), and an inability to obtain a full orthopedic evaluation without a radiograph (*n =* 3; 2%).

**Table 4.** Positive and negative experiences from early telemedicine patient experiences.

	*n*	%
What positive things did you experience during the virtual consultation?		
Less anxiety and stress related to traveling to the clinic, navigating the hospital, etc.	82	50.0
Feeling more at ease and in control being in a familiar environment	54	32.9
Longer appointment time	16	9.8
Opportunity for the physician to assess your home environment and how it may affect your recovery	13	7.9
What negative things did you experience during the virtual consultation?		
Difficulty addressing concerns or symptoms in the absence of an in-person physical examination	28	17.1
A sense of decreased interpersonal connection with your physician	20	12.2
Technical difficulties that disrupted the visit	14	8.5
Shorter appointment time	6	3.7
Inability to obtain a radiograph	3	1.8

## Discussion

In the early phases of the COVID-19 pandemic in New York City, we sought to (1) evaluate early patient experiences with our telemedicine platform for outpatient arthroplasty visits and (2) identify strengths and weaknesses of the telemedicine experience from the patient’s standpoint. We noted high levels of overall satisfaction for a variety of visit reasons and identified several positive aspects of the patient’s telemedicine experience. However, we also identified multiple areas for improvement, with the lack of an in-person examination, a decreased sense of interpersonal connection, and technical issues being the most commonly cited.

There are multiple limitations to this study. First, this is a cross-sectional survey with a heterogeneous patient population. Although most patients had never had a telemedicine visit before, approximately one quarter had. Another potential limitation is that the reasons for the visit varied widely and included preoperative, postoperative, new patient, and follow-up visits for different surgeries. These factors may be differentially associated with satisfaction and experience with the telemedicine visit. Although a more homogenous population may be helpful, the heterogeneity of this population likely reflects most arthroplasty practice and may therefore be more generalizable. Furthermore, the survey provided example prompts of positive and negative takeaways, in addition to providing open-ended free-text options. The use of example prompts may detract from the patient’s ability to provide open-ended takeaways related to their experience. Another key limitation of this study is that it focused on patient responses to telemedicine but did not evaluate the surgeon experience with the telemedicine visit. Surgeons’ responses to the telemedicine visits may shed light on how important clinical decisions (e.g., indicating patients for surgery, performing diagnostic workups) are made in the absence of an in-person physical examination. Last, as most in-person appointments were canceled or converted to telemedicine visits in the early phases of the pandemic, we did not compare these telemedicine visits with in-person visits.

Although telemedicine practices have existed for decades, with modern telemedicine consultation practices emerging as early as the 1970s to improve rural health care [6], the COVID-19 pandemic rapidly accelerated the adoption and expansion of telemedicine in orthopedics and other specialties [8,16]. Several orthopedic groups have recently described their early experiences with telemedicine programs during the COVID-19 pandemic [7–9,11,21]. Of note, most of these reported orthopedic providers’ perceptions of telemedicine, while few [7,19,21] reported patients’ perspectives. In a study of a postoperative upper extremity telemedicine program during the pandemic, Grandizio et al [7] found that telemedicine resulted in high levels of patient satisfaction, decreased visit times, and decreased travel burdens compared with conventional in-person appointments. In addition, Rizzi et al [19] surveyed 299 patients participating in telemedicine visits for a general orthopedic clinic and found high rates of satisfaction, perceived surgeon sensitivity to patient needs, and a willingness to participate in subsequent telemedicine encounters. These findings are aligned with those from the present study in an outpatient arthroplasty context.

In an effort to identify positive aspects of the telemedicine experience as well as areas for improvement, we identified several positive takeaways. In particular, patients highlighted a reduction in costs, less anxiety and stress related to travel, and feeling more at ease in a familiar environment. However, several negative experiences were also identified. First, patients expressed concern about the lack of a physical examination leading to an incomplete evaluation. Indeed, this is an important shortcoming in telemedicine. Several authors have proposed physical examination strategies that can be performed at a distance to help remedy this problem [12,15,22]. Another shortcoming noted by many patients was a sense of decreased interpersonal connection with the physician. Although this is a challenging limitation, the orthopedic provider does have some degree of ability to modulate the patient’s perceived connection with the provider. A systematic review of clinician behaviors in telemedicine identified several important practices in telehealth to preserve the physician-patient relationship, including being cognizant of differences in the pace and type of discourse, relying on visual cues to communicate empathy and build rapport, and respecting confidentiality and privacy in care delivery [10]. The third most commonly cited limitation of the telemedicine visit in the present study was technical issues that interfered with the normal visit. Examples of technical issues included having a link that only worked if the patient was logged into the hospital’s online platform, the link working on some devices but not others, audio not working, and having a narrow camera angle that made it challenging for the patient to demonstrate their lower extremities to the surgeon. Patients provided suggestions for how to address their technical issues including having a better Help Desk or having a Help Desk specifically for telemedicine, having more comprehensive instructions for installation of the telemedicine videoconferencing application, and making the logon process less complicated. These technical issues are likely multifactorial and should be addressed quickly by the clinical and information technology teams. Advanced planning and coordination by the physician’s office to ensure a streamlined visit can be helpful in this respect. Last, some patients emphasized how their telemedicine visit precluded the review of radiographs. Although some patients had undergone radiographs prior to the visit which were uploaded and reviewed during the visit, not all patients did this to minimize unnecessary exposure in outpatient satellite imaging facilities. The decision to obtain a radiograph was made by the surgeon on a patient-specific basis, balancing the need for radiographic evaluation with the need to minimize exposure during the pandemic.

In conclusion, our study of outpatient arthroplasty telemedicine visits showed high rates of patient satisfaction. Most patients noted decreased costs, with the most common increased comfort, and less travel-related anxiety associated following early telemedicine visits during the COVID-19 pandemic. We also identified several areas for improvement, such as the lack of an in-person examination, a decreased sense of interpersonal connection, and technical issues. These findings are similar to other recent studies of telemedicine experiences in the COVID-19 pandemic and highlight areas where other orthopedic providers may be able to improve their own telemedicine experiences.

## Supplemental Material

sj-pdf-2-hss-10.1177_1556331620972659 – Supplemental material for Telemedicine in an Outpatient Arthroplasty Setting During the COVID-19 Pandemic: Early Lessons from New York City Click here for additional data file. Supplemental material, sj-pdf-2-hss-10.1177_1556331620972659 for Telemedicine in an Outpatient Arthroplasty Setting During the COVID-19 Pandemic: Early Lessons from New York City by Samuel A. Taylor, Joseph D. Lamplot, Drake G. LeBrun, Christina Malfer, Mallory Wilson, Kaitlin M. Carroll, Victoria Wang, MS, David J. Mayman, Michael B. Cross, Michael M. Alexiades, Seth A. Jerabek, Fred D. Cushner, Jonathan M. Vigdorchik, Steven B. Haas and Michael P. Ast in HSS Journal®: The Musculoskeletal Journal of Hospital for Special Surgery

sj-zip-1-hss-10.1177_1556331620972659 – Supplemental material for Telemedicine in an Outpatient Arthroplasty Setting During the COVID-19 Pandemic: Early Lessons from New York City Click here for additional data file. Supplemental material, sj-zip-1-hss-10.1177_1556331620972659 for Telemedicine in an Outpatient Arthroplasty Setting During the COVID-19 Pandemic: Early Lessons from New York City by Samuel A. Taylor, Joseph D. Lamplot, Drake G. LeBrun, Christina Malfer, Mallory Wilson, Kaitlin M. Carroll, Victoria Wang, MS, David J. Mayman, Michael B. Cross, Michael M. Alexiades, Seth A. Jerabek, Fred D. Cushner, Jonathan M. Vigdorchik, Steven B. Haas and Michael P. Ast in HSS Journal®: The Musculoskeletal Journal of Hospital for Special Surgery
