# Supplementary material for: Telemedicine in an Outpatient Arthroplasty Setting During the COVID-19 Pandemic: Early Lessons from New York City
Source: HSS J. 2021 Feb 21;17(1):25–30. doi: 10.1177/1556331620972659 (PMC8077980; doi:10.1177/1556331620972659)
Supplement: sj-pdf-2-hss-10.1177_1556331620972659 – Supplemental material for Telemedicine in an Outpatient Arthroplasty Setting During the COVID-19 Pandemic: Early Lessons from New York City [file sj-pdf-2-hss-10.1177_1556331620972659.pdf]

HSS Telemedicine Patient Research Survey (2020)

1. Today's Date

   ____

   Example: January 7, 2019

2. Please select the surgery you had:

   Mark only one oval.

   - ⬭ Total Knee Replacement
   - ⬭ Partial Knee Replacement
   - ⬭ Total Hip Replacement
   - ⬭ Revision Total Knee Replacement
   - ⬭ Revision Total Hip Replacement
   - ⬭ New Consultation (no surgery)
   - ⬭ Follow-up (no surgery)

3. Please select your age range:

   Mark only one oval.

   - ⬭ 18-25
   - ⬭ 26-40
   - ⬭ 41-65
   - ⬭ 66-80
   - ⬭ >81

4. Please select your employment status:

   Mark only one oval.

   - ⬭ Full-time
   - ⬭ Part-time
   - ⬭ Unemployed
   - ⬭ Medical leave
   - ⬭ Retired

5. If employed, are you currently working from home?

   Mark only one oval.

   - ⬭ Yes
   - ⬭ No
   - ⬭ N/A

6. 1. Have you ever done a virtual consultation with a healthcare provider PRIOR to your experience at HSS?

   Mark only one oval.

   - ⬭ Yes - if 'yes', please answer the next question
   - ⬭ No - if 'no', please proceed to question #2

7. If you answered 'yes' to question #1, please select the circumstance that lead to a virtual consultation instead of outpatient visit:

   Mark only one oval.

   - ⬭ Provider only offers telehealth consultations
   - ⬭ Inability to travel due to illness and/or compromised mobility
   - ⬭ Unexpected circumstances that prevented you from attending an outpatient appointment
   - ⬭ Clinician's practice is very far from home (distance requiring an overnight stay, practice is in a different state etc.)
   - ⬭ COVID-19
   - ⬭ Other: ____________________

8. 2. What was the reason for your virtual appointment at HSS?

   Mark only one oval.

   - ⬭ New consultation
   - ⬭ 4 - 6 week post-operative follow-up
   - ⬭ 1 - year post-operative follow-up
   - ⬭ New issue unrelated to your surgery
   - ⬭ Physical therapy
   - ⬭ Other: ____________________

9. 3. How satisfied are you with the support you received during the transition process to telemedicine at HSS?

   Mark only one oval.

   - ⬭ Very satisfied
   - ⬭ Satisfied
   - ⬭ Slightly satisfied
   - ⬭ Slightly dissatisfied
   - ⬭ Dissatisfied
   - ⬭ Very dissatisfied

10. (Optional) Do you have any suggestions for how we may improve ways to support patients in transitioning to virtual care at this time and moving forward?

    ____

11. 4. What sort of device(s) did you use?
    Check all that apply:

    Check all that apply.

    - ☐ Phone
    - ☐ Tablet
    - ☐ Computer

12. 5. Did you require assistance from another person in order to set up the virtual consultation?

Mark only one oval.

- Yes
- No

13. 6. Did you experience technical difficulties while accessing the communication platform on your device?

Mark only one oval.

- Yes
- No

14. 7. Did you experience technical difficulties at any point during the virtual visit?

Mark only one oval.

- Yes
- No

15. 8. How were image and/or audio quality throughout the virtual visit?

Mark only one oval.

- Very good
- Good
- Fair
- Poor
- Very poor

16. 9. What positive things did you experience during the virtual consultation?
Check all that apply:

Check all that apply.

- [ ] Feeling more at ease and in control being in a familiar environment
- [ ] Less anxiety and stress related to traveling to the clinic, navigating the hospital, etc.
- [ ] Opportunity for the physician to assess your home environment and how it may impact your recovery
- [ ] Longer appointment time
- [ ] None of the above

17. 10. What negative things, if any, did you experience during the virtual consultation?
Check all that apply:

Check all that apply.

- [ ] Shorter appointment time
- [ ] A sense of decreased interpersonal connection with your physician
- [ ] Difficulty addressing concerns or symptoms in the absence of a in-person physical exam
- [ ] Technical difficulties that disrupted the visit
- [ ] None

18. (Optional) Please tell us more about your personal experience and how it may be impacted by the transition to telemedicine.

____

____

____

____

____

19. 11. Did you notice a reduction in expenses using telemedicine versus outpatient care at HSS?

Mark only one oval.

- Yes - if 'yes', please answer the following question
- No - if 'no', please proceed to question #12

20. If you answered 'yes' to question #11, which of the following categories were associated with any cost savings?
Check all that apply:

Check all that apply.

- [ ] Travel
- [ ] Work
- [ ] Medical

Other: [ ] _______________________________

21. 12. Compared to in-person visits, how would you rate your personal engagement and attentiveness to your own health and recovery?

Mark only one oval.

- Much better
- Better
- Same
- Worse
- Much worse

22. 13. Did you find an increase in convenience and flexibility with virtual follow-up care compared to outpatient treatment?

Mark only one oval.

- Yes
- Neutral
- No

23. 14. Did you experience the same degree of attention and interaction with your physician as you would expect in the exam room?

Mark only one oval.

- Yes
- Neutral
- No

24. 15. Do you feel that you were able to discuss all or most of your concerns during the consultation?

Mark only one oval.

- Yes
- Neutral
- No

25. 16. Did you receive rehabilitation or other services via telemedicine at HSS?

Mark only one oval.

- Yes
- No

26. 17. Overall, compared to standard outpatient treatment, how would you describe your telemedicine experience?

Mark only one oval.

- Best imaginable
- Excellent
- Good
- OK
- Poor
- Awful
- Worst imaginable

27. 18. Would you consider continuing telemedicine care in addition to outpatient treatment at HSS?

Mark only one oval.

- Yes
- Maybe
- No

This content is neither created nor endorsed by Google.

Google Forms
